# Adaptive and sequential cancer therapies emerge from treatment schedule optimization

**DOI:** 10.21203/rs.3.rs-9695978/v1

**Published:** 2026-05-29

**Authors:** Charles D. Kocher, Joseph O. Deasy, Damon R. Reed, Larry Norton, Corey Weistuch

**Affiliations:** Memorial Sloan Kettering Cancer Center, New York, NY, USA

## Abstract

Most cancer chemotherapy is delivered at the maximum tolerated dose (MTD) as often as possible, switching to a new combination of drugs upon progression. Clinical trial results have demonstrated that this sequential MTD schedule is not optimal for every patient in every situation; however, there is no systematic program for discovering what the optimal schedule actually is. We developed a simulated-annealing-based algorithm for computing personalized dosing schedules that best achieve treatment objectives. Given a mathematical model of a patient’s drug response, the algorithm returns the optimal schedule for either minimizing tumor burden or maximizing progression-free survival. We tested this algorithm on a simple mathematical model of patient treatment response; we found that there are only three optimal one-drug schedules: continuous therapy and two distinct adaptive therapies. For two drugs, each schedule used in the clinic was found to be optimal for some patient parameters, and the algorithm was able to quantitatively map out the boundaries between these schedules. The optimization algorithm developed here can play a key role in upcoming personalized cancer treatment pipelines.

## I. INTRODUCTION: A BRIEF SURVEY OF CANCER DRUG SCHEDULING

The prevailing paradigm in cancer chemotherapy treatment is that of dose-dense treatment at the maximum tolerated dose (MTD) [1–7]. In the adjuvant setting, the patient is given a fixed number of treatment cycles, generally followed by observation to detect relapse. In the non-resectable and relapse settings, patients are given sequential cycles of (combinations of) drugs until either unacceptable toxicity or disease progression occurs, at which time the next drug combination in the sequence is used (sequential MTD).

Increasingly, there have been calls to re-examine the MTD paradigm as the default in every case of cancer treatment, both in terms of the dose used per cycle and the scheduling of those cycles. For example, MTD dosing as a guiding principle was carried over by default into the realm of targeted cancer therapies, but there is limited evidence that the maximum dose of a targeted agent is the one that best balances treatment efficacy and toxicity [2, 3, 8, 9]. On the scheduling side, MTD has also been challenged by proponents of evolution-informed treatments [1, 10–12], particularly for relapsed or metastatic patients. The evolutionary argument against MTD is that maximum drug dosing applies the maximum selective pressure for resistance, so that MTD scheduling hastens the development of resistance and ultimately progressive disease. This perspective is bolstered by recent positive clinical experience with adaptive therapy [13], which showed that introducing response-informed treatment holidays increased progression-free survival (PFS) of metastatic castrate resistant prostate cancer patients beyond the PFS achieved by using MTD (note, however, that the drug used in this study was abiraterone—a hormone therapy—not a cytotoxic chemotherapy).

**FIG. 1.**
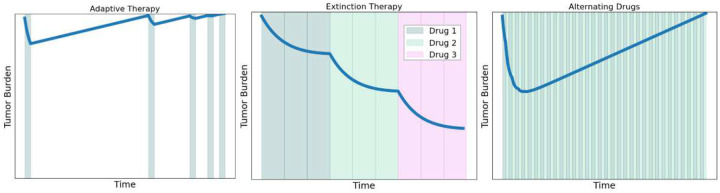
Some proposed alternatives to sequential MTD scheduling. As discussed in the text, these schedules are motivated by evolutionary theory [14–16], and they have differing levels of clinical testing and implementation.

Using mathematical models of treatment response, many alternative treatment schedules have been developed that seek to replace MTD in different scenarios (see Fig 1); these schedules are being tested in the clinic. Above, we mentioned adaptive therapy [13, 14, 17], which aims to prolong PFS. There are currently clinical trials in basal cell carcinoma (NCT05651828), melanoma (NCT03543969), metastatic castrate resistant (NCT05393791, NCT06216249) [18] and sensitive (NCT03511196, NCT06734130) prostate cancer, and ovarian cancer (NCT05080556). Extinction therapy [1, 15, 19] aims to cure patients using a well-timed sequence of therapy changes (successive “strikes”). It is currently being evaluated in rhabdomyosarcoma (NCT04388839) [20], Ewing sarcoma (NCT07194044) [21, 22], metastatic castration sensitive prostate cancer (NCT05189457), and metastatic breast cancer (NCT06409390). In addition, the standard of care treatment in acute lymphoblastic leukemia is an extinction-like therapy, although the exact sequencing of drugs was discovered empirically, not through modeling [10, 23]; similarly for the chemotherapy regimen AC-T (sequential administration of a taxane like paclitaxel and combined doxorubicin and cyclophosphamide) used in the adjuvant setting to treat localized breast cancer, where the treatment is switched without any sign of progression [24–26].

These alternative schedules hope to improve patient outcomes, decrease toxicities, and lower treatment costs. Caution is warranted, however. It has been demonstrated that intermittent treatment does not always mean higher quality of life, even though toxicity is lower [27]. Modeling incorporating evolutionary principles has tried to predict better treatment schedules before with limited success. For example, in [16], it was proposed that alternating cycles of non-cross resistant drugs would give the best outcome^1^. When this alternating strategy was tested in the clinic, a sequential schedule proved significantly better than the alternating schedule [28]; sequential MTD prevailed. In spite of this notable failure, alternating schedules are still the standard of care for Ewing sarcoma (VDC/IE) [29, 30], osteosarcoma (MAP) [31, 32], and Burkitt lymphoma (CODOX-M/IVAC, hyperCVAD + R) [33–35], as well as finding use in germ cell tumors (TI-CE) [36], pancreatic cancer (nab-P/Gem-mFOLFOX) [37], and Wilms tumor (e.g. DD4A and Regimen M, among others) [38–44].

In summary, improving on the sequential MTD paradigm is an achievable goal, but the difficulty of the problem, as evidenced by the paucity of clinical trial results that go against sequential MTD, should be appreciated. Breakthroughs have been made, but current results are limited. Moreover, the alternative schedules (adaptive therapy, extinction therapy, alternating therapies) that have been proposed and are currently being tested in or are already in use in the clinic are based on heuristics, leaving room for further optimization and personalization [17, 45–51]. In order to maximize the chances of success, it would be ideal to know that a proposed schedule is in some way optimal before undertaking a clinical trial.

To address this need, we have developed an algorithm based on simulated annealing for finding the optimal drug schedule—either for minimizing tumor burden or maximizing PFS—given a reasonable model of a patient’s treatment response. We used this algorithm to probe the optimal schedules of a simple mathematical model of patient treatment response which was proposed previously in [52], creating “phase diagrams” of what schedules would be optimal for which types of patients. We found that, when only one drug was available, there were only three classes of optimal schedules, including the adaptive therapy schedule we discussed above. For two drugs, the sequential MTD, alternating, and second-strike extinction therapy schedules were all found as optimal schedules in different parts of the patient parameter space. The algorithm we developed can play a key role in personalizing patient treatment decisions once the rest of the personalization pipeline (better modeling and prediction of patient treatment response) has been developed.

## II. METHODS

### A. Using simulated annealing as the basis for the schedule optimization algorithm

First, we formulate our schedule optimization problem. To make the problem tractable, we cut time up into segments of one week. During each week, patients can receive one of *N* treatments^2^, or can receive no drug (denoted by 0). Therefore, a treatment schedule *C* is a sequence of numbers drawn from the set {0,1,2,…,N}.

We assume that a mathematical model of the patient’s response to all treatments is known. In our applications below, this assumption means that we know all of the parameters that go into an ODE model of tumor burden for a patient before beginning treatment. Estimation of these parameters a priori is beyond the scope of this manuscript and is the subject of ongoing research.

Given a model of treatment response and some initial conditions of that model, the drug schedule *C* can be used to simulate the patient’s tumor burden trajectory, *T*(*t*;*C*). The simulation should continue until either cure is reached (during the drug dosing period, by dropping the tumor burden below a set cure threshold *T*_cure_), or progression is reached (by the tumor burden crossing a set progression threshold *T*_prog_). If cure is not reached, then the treatment schedule can be padded with 0’s until progression is reached. Therefore, each simulated trajectory ends at either *T*_cure_ or *T*_prog_.

Next, we quantitatively defined the objectives we wanted to optimize. The first treatment objective is curing the cancer. The optimal curative schedule was assumed to be the one that reached the lowest tumor burden level at any point of its trajectory (i.e. the one that was closest to *T*_cure_). We introduced an energy landscape,

(1) $${ℰ}_{\text{cure}}\left(C\right)=\text{log}\left[1+\underset{t}{\text{min}\;}T\left(t;C\right)\right],$$

which gives the optimal curative schedule when minimized over all schedules *C*. The second treatment objective we considered was maximizing the PFS. We similarly introduced an energy landscape^3^,

(2) $${ℰ}_{\text{PFS}}\left(C\right)=\frac{100}{\text{log}\left[1+\underset{t}{\text{min}}\left\{t:T\left(t\right)\geq T_{\text{prog}}\right\}\right]},$$

which gives the optimal PFS schedule when minimized over all schedules *C*.

Simulated annealing can be used to find the global minima of complicated energy landscapes [53, 54], like the ones in Eqs (1) and (2). We now sketch how we applied it to the schedule optimization problem. First, a schedule *C* was initialized and the energy *ℰ* was calculated. Then, a change was made to the schedule (either switching one of the numbers in the sequence *C* to one of the *N* other numbers, or moving a non-zero number to the left or right if the target space was a zero) to produce a test schedule *C*′ with corresponding energy *ℰ′*. If *ℰ′ < ℰ*, the change is accepted. If *ℰ′* > *ℰ*, then the change is probabilistically accepted with probability

(3) $$P\left(\text{accept},n\right)=\text{exp}\left[-\beta \left(n\right)\left({ℰ}^{\prime }-ℰ\right)\right].$$

where *n* is the number of iterations performed so far and *β*(*n*) is the “cooling schedule” (an increasing function of *n*, since it is the inverse temperature) that ensures the algorithm converges to the minimum of *ℰ*. We used

(4) $$\beta (n)=\left\{\begin{array}{ll} 0.01 & n<n_{\text{max}}/4\\ 0.01\;\text{exp}\left[10\left(n/n_{\text{max}}-1/4\right)\right] & n\geq n_{\text{max}}/4 \end{array},\right.$$

with *n*_max_ = 10,000 being the number of iterations of the simulated annealing algorithm, throughout, for both minimizing the tumor burden and maximizing PFS.

For our specific application, *ℰ′ = ℰ* is a special case: Not all schedules that have the same energy are actually equal. For example, if cure is achieved in both schedules (say at *t*_cure_ and $t_{\text{cure}}^{\prime }$, the schedule which cures first should be preferred. In this case, the acceptance probability was set to $P(\text{accept})=\text{min}\left\{t_{\text{cure}}/t_{\text{cure}}^{\prime },1\right\}$ (if only one schedule cured, that schedule was definitely accepted). Similarly, if two schedules had the same *ℰ*, but used different amounts of drug, the schedule that minimizes the amount of drug used was preferred. In this case,

(5) $$P(\text{accept})=\text{min}\left\{\frac{1+|\{c\in C:c>0\}|}{1+\left|\left\{c\in C^{\prime }:c>0\right\}\right|},1\right\},$$

where | · | denotes the cardinality of a set.

**FIG. 2.**
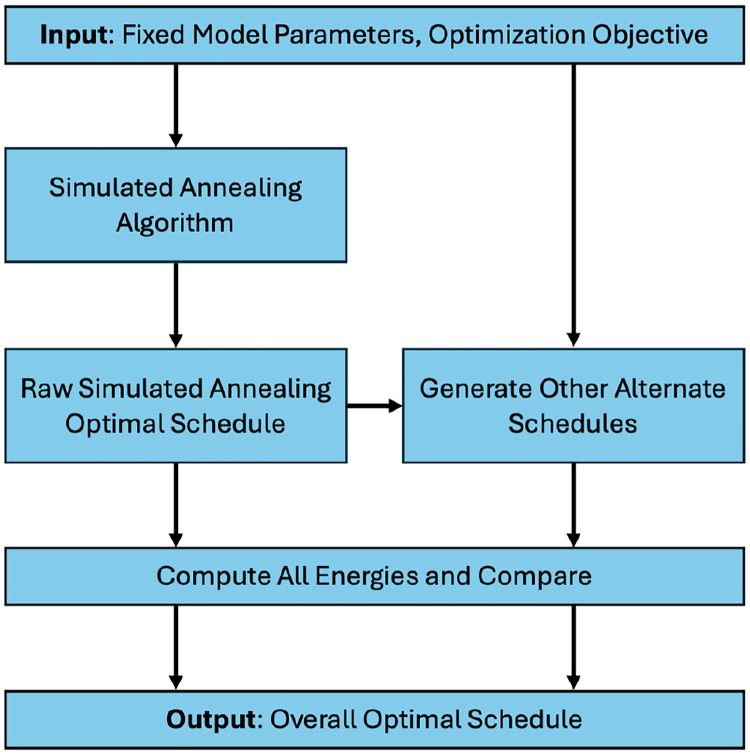
Schematic of the schedule optimization algorithm. Given a mathematical model of drug response, a patient’s parameters in that model, and the treatment objective (minimize tumor burden or maximize PFS), the algorithm outputs the schedule that optimizes that treatment objective for that patient in that model.

As discussed in SI Appendix B, we tested the simulated annealing algorithm by comparing its output to a few cases where the global optimal schedule could be found by brute-force enumerating all possible schedules. We observed some noise and variability around the optimal schedule when the total required treatment time was long. While this variability had little impact on the performance of the algorithm (i.e. the PFS times or minimum tumor burdens achieved by the raw simulated annealing output schedule were always very close to optimal), the theoretical interpretation of the model’s output, e.g. in our phase diagrams below, could be distorted by it. To reduce this noise, we added a comparison of the simulated annealing algorithm’s raw output to a few other well-motivated schedules, then picked the best of these as the optimum. For example, for one drug, we additionally tested a continuous dosing schedule, an adaptive therapy schedule, and a “standardized” version of the simulated annealing output. More details can be found in SI Appendix B. A full schematic of our optimization algorithm can be seen in Fig 2.

### B. The GDRS model is a simple but realistic description of patient treatment response

Our optimization algorithm requires a model that predicts patient treatment response and an individual patient’s parameter values in that model. To test the algorithm, we used the GDRS model of [52]:

(6a) $$\frac{dT}{dt}=\left(\gamma -\sum_{i}\delta_{i}D_{i}\left(t\right)E_{i}\left(t\right)\right)T\left(t\right),$$

(6b) $$\frac{dE_{i}}{dt}=\left[s_{i}\left(1-E_{i}(t)\right)\left(1-D_{i}(t)\right)-r_{i}D_{i}(t)\right]E_{i}(t),$$

where *T*(*t*) is the tumor volume (or proxy for it) at time *t* and *D*_*i*_(*t*) ∈ [0, 1] is the dose of drug *i* (scaled by the MTD). The GDRS model is a minimal model describing tumor Growth (rate *γ*), Death due to drug (rate *δ*_*i*_), Resistance to drug (rate *r*_*i*_), and re-Sensitization to drug (rate *s*_*i*_). It is a phenomenological model, via the use of the “drug efficacy” parameter *E*_*i*_(*t*) ∈ [0, 1] to quantify the degree of resistance in the tumor population.

A similar model was derived from first principles in [55] that connects the terms in GDRS-like models to the mechanisms from which they arise and clarifies the GDRS model’s implicit assumptions. GDRS assumes that tumors grow exponentially, with no difference in growth rate between the sensitive and resistant cells when there is no drug. Drug action/resistance mechanisms are assumed to be independent, so that the population fractions of sensitive and resistant cells act as independent probabilities. For example, if *p*_*k*_ is the fraction of cells sensitive to drug *k*, then *p*_1&2_ = *p*_1_*p*_2_. This type of independence has been used before to accurately model treatment response [56].

For our simulations, we used the GDRS model with the initial condition *T*(0) = 1×10^12^ cells (this starting point was also used in [56]) and *E*_*i*_(0) = 1 for all drugs *i*. The progression threshold was set to *T*_prog_ = 2*T*(0) = 2×10^12^ cells so that an untreated tumor would progress in one doubling time, and the cure threshold was set to *T*_cure_ = 1 cell.

To demonstrate the utility of this model beyond the examples shown in [52], we have given an example in SI Appendix A of how it fits clinical data from MSK-CHORD [57]. Given the limited resolution of cancer patient data, we conclude that, while simple, the GDRS model should give a reasonable representation of the space of patient drug responses, so that the optimal schedules we compute using it will give a reasonable representation of the space of optimal strategies.

## III. RESULTS AND INTERPRETATION

### A. There are only three types of optimal schedule for one drug.

**FIG. 3.**
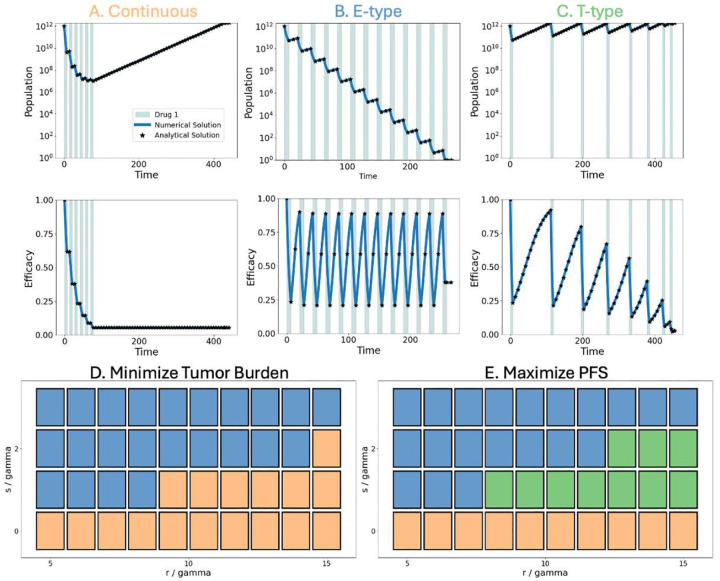
Phase diagrams for optimal schedules with one drug. There are three types of optimal schedule for one drug, shown here with their effects on cancer population (first row) and drug efficacy (second row): (A) Continuous therapy, where drug is used every possible window. (B) *E*-type adaptive therapy, where drug is used when an efficacy threshold is crossed. (C) *T*-type adaptive therapy, where drug is used just before progression. (D and E): Results of sweeping through *r*-*s* phase space at constant *γ* and *δ* and finding the optimal schedule. Color on the phase diagram corresponds to the color of the titles for (A-C).

After formulating the algorithm, we explored the GDRS parameter space for one drug. Through this non-systematic searching, we noticed that the three types of schedules^4^ in Fig 3(A–C) were the optimal ones. One was continuous treatment, where drug was used every time possible. The other two were types of adaptive therapy, which we have called *T*-type and *E*-type after the parameters of the GDRS model that mainly inform the strategy.

In *E*-type adaptive therapy, one window of treatment is followed by a treatment break that lasts until the drug efficacy *E* reaches some acceptable level (via re-sensitization), at which point drug is used again. These schedules are largely characterized by cycles in which *E* returns to its original value, but *T* ends up lower. This mechanism and the trajectories produced by it are reminiscent of radiotherapy, in which treatment breaks via fractionation allow for cell cycle redistribution and other mechanisms of re-sensitization [58].

In *T*-type adaptive therapy, a window of treatment is followed by a treatment break that lasts until the tumor burden *T* approaches the progression threshold, at which point drug is used again to prolong PFS. The idea of this schedule is to use drug only when necessary so that sensitivity can build up in between doses. This type of adaptive therapy is close to, but not exactly the same as, the adaptive therapy introduced in [13, 14], because here we do not set a response threshold for stopping treatment. We only use the progression threshold for turning the drug on.

Our heuristic understanding from anecdotal examples was that these three schedules were the only ones that could be optimal for one drug. We fixed the GDRS parameters *γ* (growth) and *δ* (drug kill) and swept over resistance-sensitivity *r*-*s* parameter space, finding the optimal schedule at each point. We were then able to sort the obtained optimal schedules into the three types we identified above using the schedules we tested for the optimum (see SI Appendix B): The continuous schedule was identified with the continuous strategy, the adaptive therapy schedule with the *T*-type adaptive therapy strategy, and both the raw and standardized simulated annealing output were mapped to the *E*-type strategy.

The resulting phase diagrams for both objectives, minimizing tumor burden and maximizing PFS, are shown in Fig 3(D–E). Except for three points on the phase boundary, the PFS phase diagram is the same as the tumor burden phase diagram but with the *s* > 0 continuous strategies turned into *T*-type adaptive therapy. The three exception points turned *E*-type adaptive therapy into *T*-type adaptive therapy. In Appendix C, we investigate these points, showing that the anomalous behavior happens due to the discretization of time into cycles. Accordingly, the phase boundary here acts like a second-order phase transition, where properties of both the *T*-type and *E*-type schedules can emerge near the boundary, for both optimization objectives.

### B. For two drugs, the clinically used schedules are optimal for some parameters

**FIG. 4.**
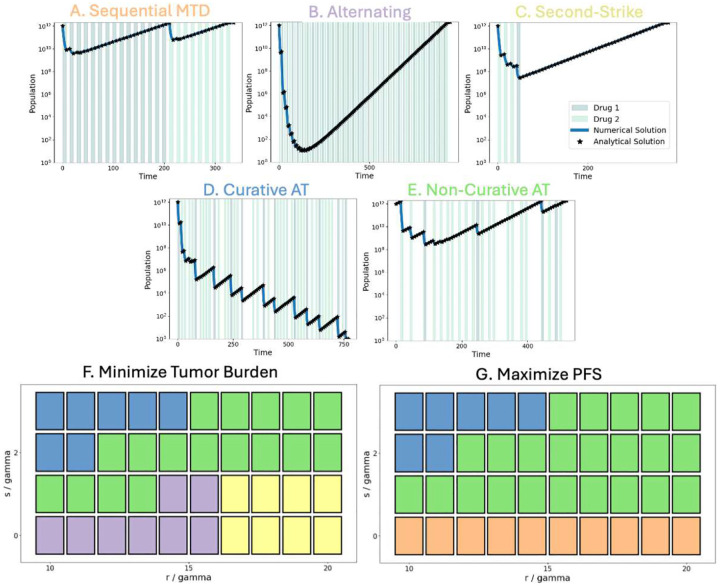
Example phase diagrams for the two drug case. The simulated annealing algorithm returns the schedules of Fig 1 as optimal for different parts of parameter space: (A) sequential MTD, (B) alternating drugs, (C) second-strike. Additionally, (D) curative and (E) non-curative adaptive therapies analogous to the *E*- and *T*-type, respectively, adaptive therapies for one drug were found to be optimal. (F and G) Sweeping through resistance-sensitivity phase space of the first drug. Coloring corresponds to the titles for (A-E). For more details on the adaptive therapy schedules, see SI Appendix C.

We next tested various parameters for two drugs. As in the single drug case, we first explored parameter space by choosing different parameter values in an ad hoc manner. We were able to confirm that clinically used schedules (sequential MTD, alternating, and second-strike) were the optimal schedule for some parameter values. Some of the optimal schedules we obtained are shown in Fig 4(A–E).

The full phase space for two drugs is vast; instead of doing a systematic analysis as for one drug, we chose a few different ranges of parameter values for the first drug and created phase diagrams; see Fig 4(F–G). We were able to capture phase boundaries between pairs of the alternating, second-strike, sequential MTD, and adaptive therapy strategies. The optimal schedules that we identified as two drug adaptive therapies could again be sorted into two different buckets, curative and non-curative, in direct analogy to the *E*- and *T*-type adaptive therapies of Fig 3. Further discussion of this comparison is given in SI Appendix C, as the “second-order-like” phase transition boundary becomes more pronounced in the two drug case.

## IV. DISCUSSION

In this paper, we have reported two main results. First, we have created a simulated-annealing-based algorithm for finding a patient’s personalized optimal treatment schedule given that a way to model the patient’s response to each possible treatment is known. This algorithm was validated in simple cases, and it can be easily adapted to use any mathematical model of patient treatment response. Second, we used the algorithm to explore the space of optimal schedules in the simple GDRS model of cancer patient treatment response. For one drug, we mapped out the phase diagram of optimal schedules and found only three different types of schedules could be optimal: continuous therapy, *T*-type adaptive therapy, and *E*-type adaptive therapy. We found features similar to those of a second-order phase transition at the boundary. For two drugs, we found that each of the clinically used schedules was optimal for some parameters and gave examples of the phase diagrams, illustrating the boundaries between these schedules.

These latter results all depend on the GDRS model. In principle, if we were to use a different treatment response model in our optimization algorithm, then they could change. However, as we argued in Section IIB and SI Appendix A, the GDRS model is a reasonable description of actual patient treatment responses. Therefore, the results that we report here should have a similar degree of generality: optimal schedules using other models with independently-acting drugs and U-shaped treatment response trajectories (ones that reproduce patient dynamics such as in Fig S1) should have similar features.

The optimization algorithm was able to resolve the precise phase boundaries between these strategies, which had not previously been achieved. Our phase diagram approach leads to an important observation: Different patients in the same clinical context could fall on different sides of these phase boundaries (because of the intrinsic characteristics of their disease, which manifest as different GDRS parameters). For example, in a hypothetical clinical trial where this situation arises, the best results could be achieved only by personalizing the patients’ strategies. A “one-schedule-fits-all” approach would always be sub-optimal. Predicting where patients fall on the phase diagram ahead of time using baseline demographic, clinical, genomic, or other data (e.g. generalizing successes in predicting radiotherapy response [59–61]), or from early treatment response [62, 63], is an open problem; the combination of machine learning and large-scale clinical data aggregation [57] is a promising potential route toward a solution.

We also found that sequential MTD was optimal for maximizing PFS when the re-sensitization parameter of each drug was low. This result should serve as another reminder that improving upon the sequential MTD paradigm could be an incredibly difficult problem, as we highlighted in Section I. It very well could be the case that many of our patients and treatments are in the region where sequential MTD is actually optimal, so that improvement is not possible (a similar argument could explain why alternating therapy failed in a breast cancer trial [7, 28]). On the other hand, for situations where sequential MTD is not the clinically used schedule, it is possible that these patients, tumors, and drugs are in a different region of parameter space. However, we must also consider explanations drawing on effects beyond those of the simple GDRS model, such as collateral sensitivities [64–66] or pharmacodynamics.

## V. CONCLUSION

So far in clinical practice, testing alternatives to the sequential MTD scheduling paradigm has been a patchwork that has produced notable successes and failures. We have introduced an algorithm for finding the optimal cancer treatment schedule given a mathematical model of a patient’s response to each possible treatment and demonstrated the types of optimal schedules that it can find using the simple GDRS model. Once reliable methods for predicting (and updating in real-time) patient treatment response are implemented, such an algorithm can provide a framework for systematically optimizing the use of existing drugs through personalized scheduling.

## Supplementary Material

1

## Data Availability

Code used to create all the plots in this paper, which includes the simulated annealing optimization algorithm, can be found at https://github.com/cdkocher/optimal-cancer-treatment-scheduling.

## Footnotes

1 Interestingly, in the same paper, they model a version of extinction therapy, foreshadowing its development many years later.

2 For simplicity, treatments are assumed to be dosed at the MTD, so that combinations are not allowed. However, treatments themselves can denote combinations of drugs. I.e., treatment 1 can be a three drug cocktail, where we assume those drugs are always used together.

3 Note that the factor of 100 in the numerator of Eq (2) was used to roughly equalize the scales of *ℰ*_cure_ and *ℰ*_PFS_ in the simulations that we performed, so that the same cooling schedule could be used for both.

4 In these results and the ones that follow, we enforced a rest week after each treatment, so that drug must be followed a 0 in the sequence. This rest week can be turned on or off. See SI Appendix D for a discussion on the guiding principles we used when choosing which parts of parameter space to explore.
