# Supplementary material for: Adaptive and sequential cancer therapies emerge from treatment schedule optimization
Source: Res Sq. 2026 May 29:rs.3.rs-9695978. Preprint. [Version 1] doi: 10.21203/rs.3.rs-9695978/v1 (PMC13232422; doi:10.21203/rs.3.rs-9695978/v1)
Supplement: 1 [file NIHPPRS9695978V1-supplement-1.pdf]

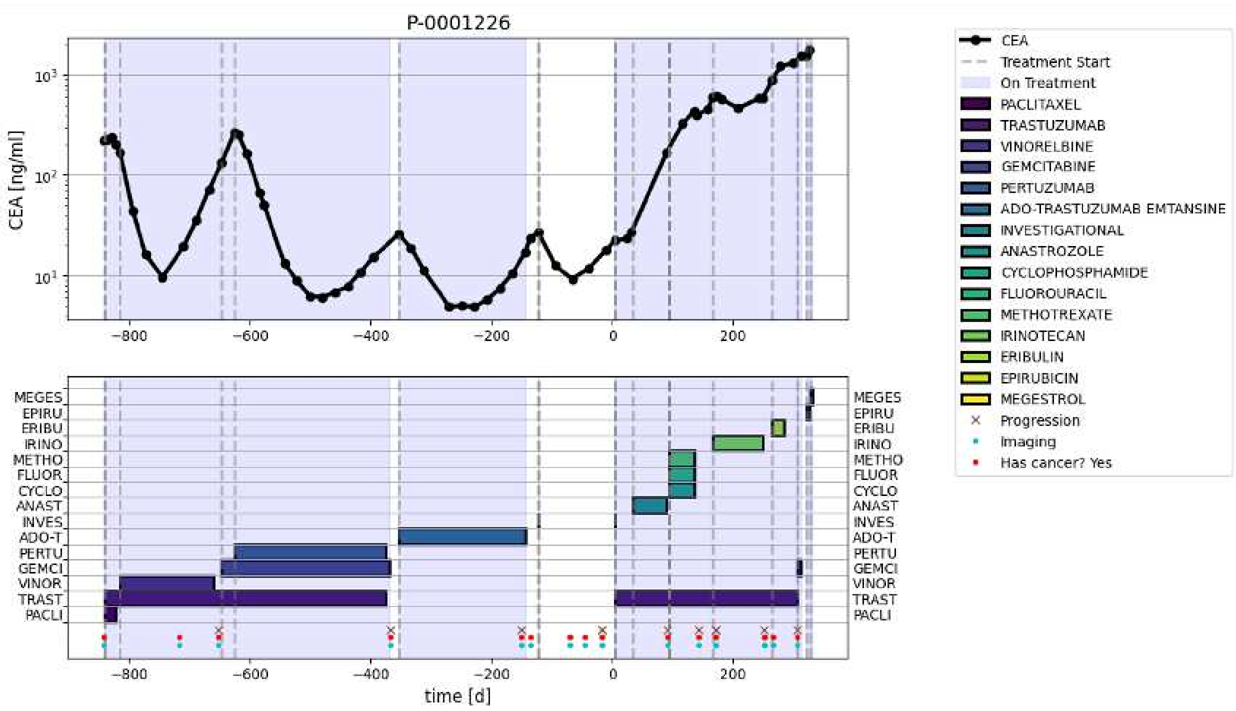

FIG. S1. **An example patient trajectory from MSK-CHORD.** This patient had metastatic breast cancer.

Appendix A: GDRS model fits data from MSK-CHORD

MSK-CHORD [57] is a dataset that has longitudinal blood biomarker data for a few cancer types, as well as treatment data and genomics. This data set can be viewed on cBioPortal. Here, we just use the clinical information, not the genomics. As an example, look at Fig S1. Shown is patient P-0001226, diagnosed at time $t = -3665$ days (t is the difference from when the first genomic sequencing was finished) with AJCC stage I (HR+, HER2+) breast invasive ductal carcinoma which ultimately metastasized, leading to the patient's death at $t = 334$ days. MSK-CHORD reports the carcinoembryonic antigen (CEA, a proxy for tumor burden in some advanced breast cancer cases) level over time for this patient, as well as all treatments, imaging results, and progression, as extracted from clinical and radiological notes.

Fig S1 shows a clear U-shaped response of the patient's CEA level to the different treatments, the characteristic sign of evolutionary rescue by drug resistance [1, 10, 67, 68]. That is, the population of cancer cells that is treatment sensitive—the bulk of the initial population—declines in number while the drug is applied, but a small resistant population—which is

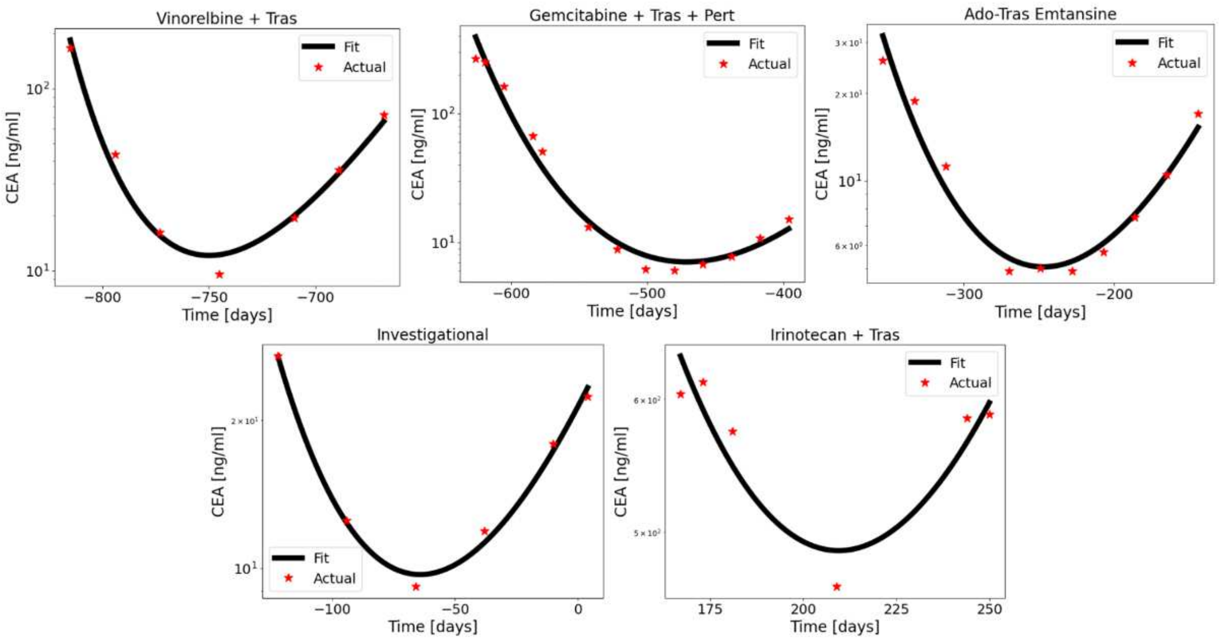

FIG. S2. **Fitting the GDRS model to MSK-CHORD patient data.** The GDRS model, Eqs (6), was fit to the five U-shaped regions of the patient CEA trajectory shown in Fig S1. Agreement between the fit and data are reasonable: The fit is able to roughly capture the timing and depth of the CEA minimum, suggesting the GDR model is capturing the essence of the CEA dynamics.

either pre-existing or discovered *de novo* through genetic or epigenetic changes—is able to continue growing. Eventually, the total population of sensitive plus resistant cells reaches a minimum, after which the population is mostly resistant cells, and treatment begins to fail. Ultimately, imaging-confirmed progression is reached (brown x on the bottom plot), prompting a switch in treatment.

We can fit the U-shaped curves from this patient using the GDR part of the GDRS model (that is, no re-sensitization because the drug is never turned back on after it is turned off). Results are shown in Fig S2. To do so, we manually picked out the treatment windows that this patient had U-shaped responses. We then estimated this patient's CEA growth rate as $\gamma = 0.042$ per day by taking the maximum growth rate implied by the difference between consecutive CEA measurements. We then used this value of γ to find δ_i and r_i for each drug by using SciPy's curve_fit function on the log of the CEA data with the analytical solution to the GDR model. The full method can be found in the code provided on GitHub (see code

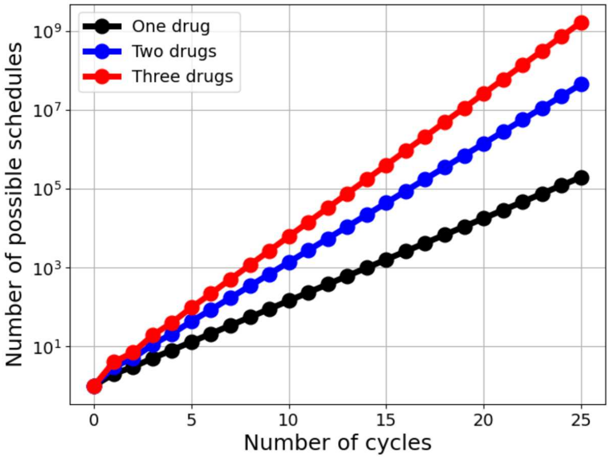

FIG. S3. **Number of schedules as a function of the number of cycles (weeks), for one to three drugs.** The scaling is exponential in the number of cycles, but searching approximately 10^4 schedules was found to run reasonably quickly on the author's laptop.

availability statement in the main text).

Appendix B: Validating the simulated annealing algorithm using brute-force enumeration and fine-tuning by testing additional schedules

We wanted to test the simulated annealing algorithm in cases where the optimal schedule was known exactly. To do so, we wrote a separate optimization algorithm that enumerated all schedules of a specified length, created their tumor population trajectories, then picked out the optimal one according to the rules used for the simulated annealing algorithm. This brute force method is tractable if the number of weeks is not too large and a rest week is enforced: see Fig S3, which shows the total number of schedules as a function of the total number of weeks for one to three drugs.

To test minimizing the tumor burden, we used $\gamma = \log(2)/20$, $\delta = 50\gamma$, $r = 6\gamma$, and $s = 45\gamma/4$. To test maximizing the PFS, we used $\gamma = \log(2)/20$, $\delta = 25\gamma$, $r = 15\gamma$, and $s = \gamma/2$. We enumerated all schedules over a 16 week period, with mandatory rest weeks after using drug. The results of the enumeration are shown as the top row in Fig S4; the simulated annealing output is shown as the bottom row.

The only difference between the figures is that we have shown the full 16 weeks in the brute force case to illustrate our next point. We now prove that the brute force enumeration

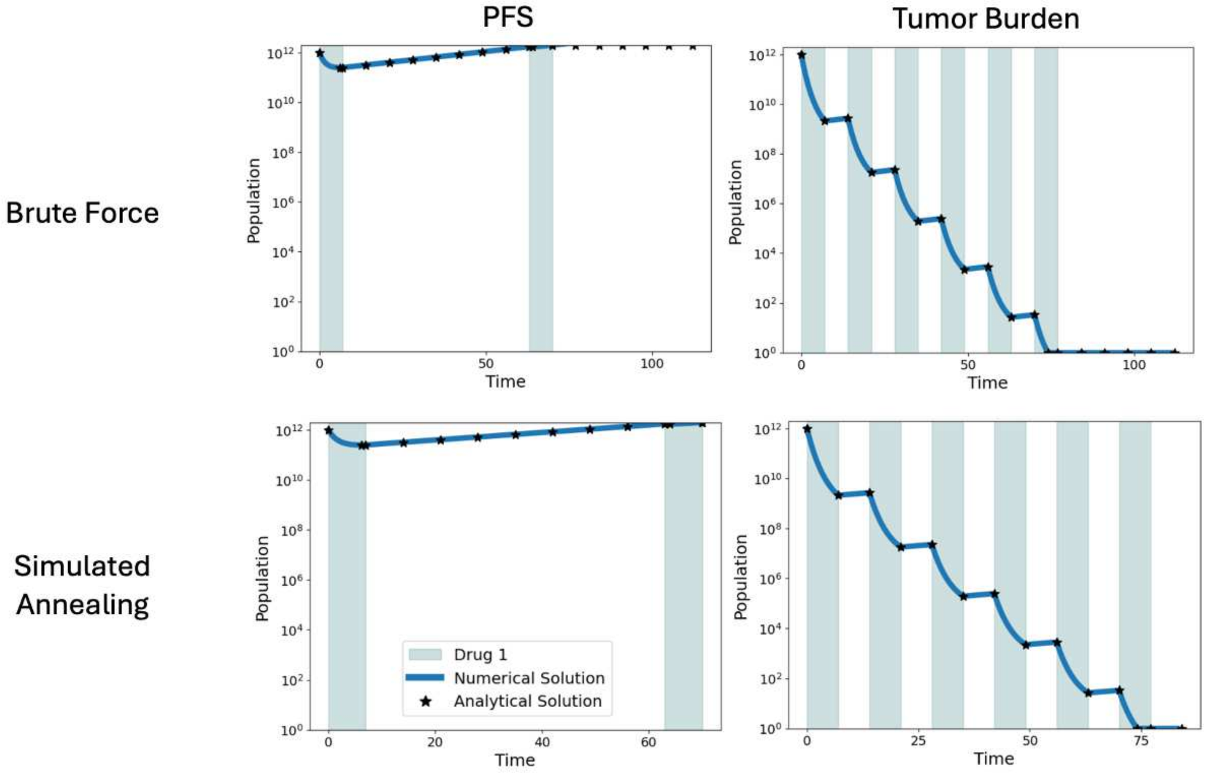

FIG. S4. **Comparing the simulated annealing algorithm to a brute force approach.** Simulated annealing gives the same optimal schedule. These schedules are their respective global optima because adding more cycles cannot impact the optimum PFS or cure time.

is finding the global optimum in each case. First, in the PFS case, all enumerated schedules progressed by the 16th cycle (the optimal schedule shown being the one that took the longest to do so). Therefore, adding cycles cannot change the optimal schedule: any treatments after the last one shown cannot impact the trajectory because the patient has already progressed. We have enumerated all potentially optimal schedules, meaning we have found the global optimum. Similarly, in the cure case, adding cycles cannot impact the optimum. The optimal schedule is the one that cures first, so only changes before the shown cure time would impact the outcome. Since all such schedules were already in the enumeration, we have found the global optimum.

However, the simulated annealing algorithm is stochastic. Accordingly, it does not always find the same optimal schedule. In the simple cases of Fig S4, there is no issue: we ran the simulated annealing algorithm 100 times on each and observed that it found the globally

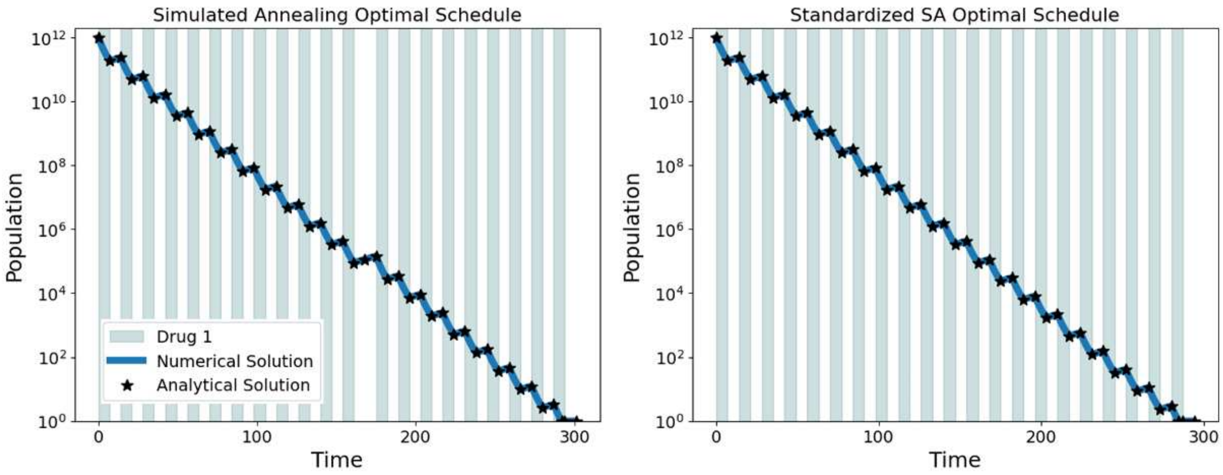

FIG. S5. **Testing a standardized version of the simulated annealing output increases the accuracy.** Simulated annealing struggles to find the global optimum (right) when the search space gets large, outputting incorrect answers like the one shown here (left) 69/100 times for these parameters. Testing a schedule that standardizes the time between drug doses increases the accuracy to 99/100 in this case.

optimal schedule all 100 times (see the code).

When the GDRS parameters are such that the optimal schedule is much longer (i.e. the space of potentially optimal schedules is much larger than the 10,000 iterations we are doing for the simulated annealing), then the stochasticity can cause variability in the output. For example, consider Fig S5. The optimal strategy for these parameters is to use drug every time available, leading to cure in 41 cycles. However, there are over 430 million schedules of this length, much more than the 10,000 iterations of the simulated annealing algorithm. Accordingly, the algorithm does not always sample the exact global optimum: for this case, it only found the correct schedule 31/100 times (see the code). As shown in the figure, the difference in cure time between the global optimum and the returned schedule was often very small; however, the interpretation of the optimal schedule is different for the left and right side of Fig S5. On the right, the true optimum is a continuous dosing schedule. On the left, the nearly-optimal schedule is not a continuous dosing schedule. Such differences can cause problems when trying to draw theoretical conclusions about optimal scheduling.

To address this issue and therefore decrease some of the stochasticity around the globally optimal schedule of the raw simulated annealing algorithm's output, we tested a small

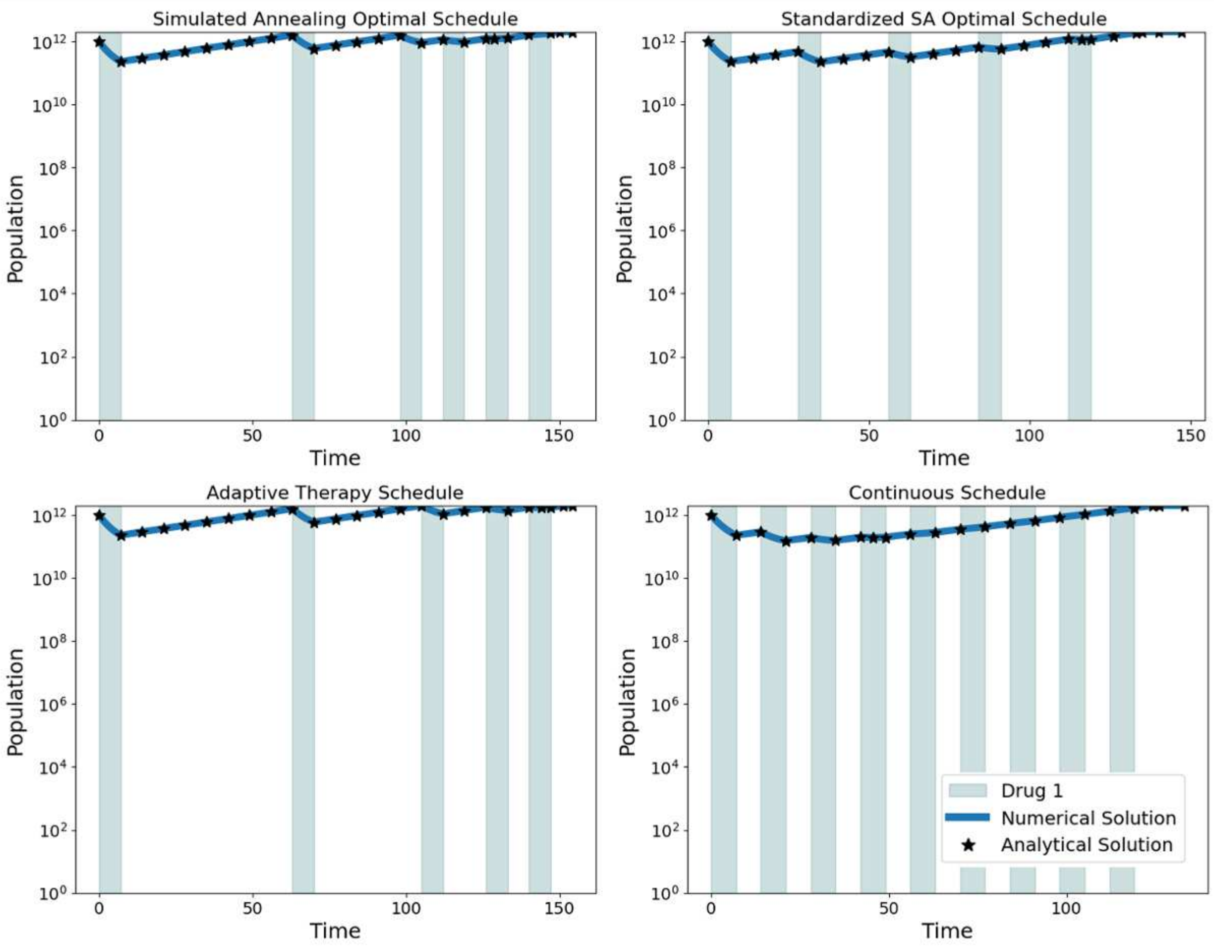

FIG. S6. **The additional tested schedules and the simulated annealing optimum for one drug.** We test the simulated annealing output against a standardized version of itself, an adaptive therapy schedule, and a continuous dosing schedule. The optimal schedule, which in this case (trying to maximize PFS) was the adaptive therapy schedule, is the one that has the lowest energy (including tiebreakers). PFS values were 252 days for the simulated annealing schedule, 230 days for the standardized SA schedule, 255 days for the adaptive therapy schedule, and 184 days for the continuous therapy schedule.

number of other schedules in addition to those sampled during the simulated annealing, as shown in Fig S6. These schedules were ones we expected to be optimal for various parameters. For one drug, they included continuous therapy (dose every available time), a form of adaptive therapy (only dose when the patient would progress the next cycle without it), and a "standardized" version of the simulated annealing output that used a constant period between cycles that matched the rounded average period between cycles of the simulated

annealing algorithm's returned optimal schedule. The schedule with the lowest energy (including tiebreakers) was then reported as the optimal schedule. To further illustrate the utility of this additional testing, for the parameters used to create Fig S5, adding just the standardization resulted in the correct optimal schedule being found 99/100 times (see the code). Explicitly testing the continuous dosing schedule would increase this accuracy to 100/100, since continuous dosing is the exact global optimum in this case.

For two drugs, there are far more heuristic schedules that could be optimal and could be separately tested for each run of the algorithm. As shown in Fig 4(A-C), we chose to test the sequential, alternating, and second-strike schedules, of both drug orders, since these schedules were the ones we are clinically interested in.

Appendix C: Further information on the phase boundaries in the one drug case

One drug. To investigate the anomalous points in Fig 3(E) that we pointed out in the main text, we needed to create analytical metrics for predicting which strategy should be optimal on the PFS phase diagram. For example, we can analytically calculate when "curative cycles" that keep E unchanged but decrease T exist for GDRS parameters (γ, δ, r, s), giving E-type trajectories that lead to ever-decreasing tumor burden. Setting $D(t) \in \{0, 1\}$, the GDRS equations for one drug are

$$\frac{dT}{dt} = \begin{cases} \gamma T \, , & \text{drug off} \\ (\gamma - \delta E)T \, , & \text{drug on} \end{cases} \, , \tag{S1}$$

$$\frac{dE}{dt} = \begin{cases} sE(1-E) \, , & \text{drug off} \\ -rE \, , & \text{drug on} \end{cases} \, . \tag{S2}$$

We want to find a cycle that starts at (T_0, E_0), applies drug for time dt_1, leaves drug off for time dt_2, and then ends up at $(T_0 + dT < T_0, E_0)$. During the first leg of the cycle, E changes by an amount $dE_1 = -rE_0 dt_1$. During the second leg, E changes by an amount $dE_2 = sE_0(1-E_0)dt_2 + O(dt^2)$. For E to return to the same value E_0 at the end of the two-step cycle, we must have $dE_1 = -dE_2$, or

$$dt_2 = \frac{r dt_1}{s(1-E_0)} \, . \tag{S3}$$

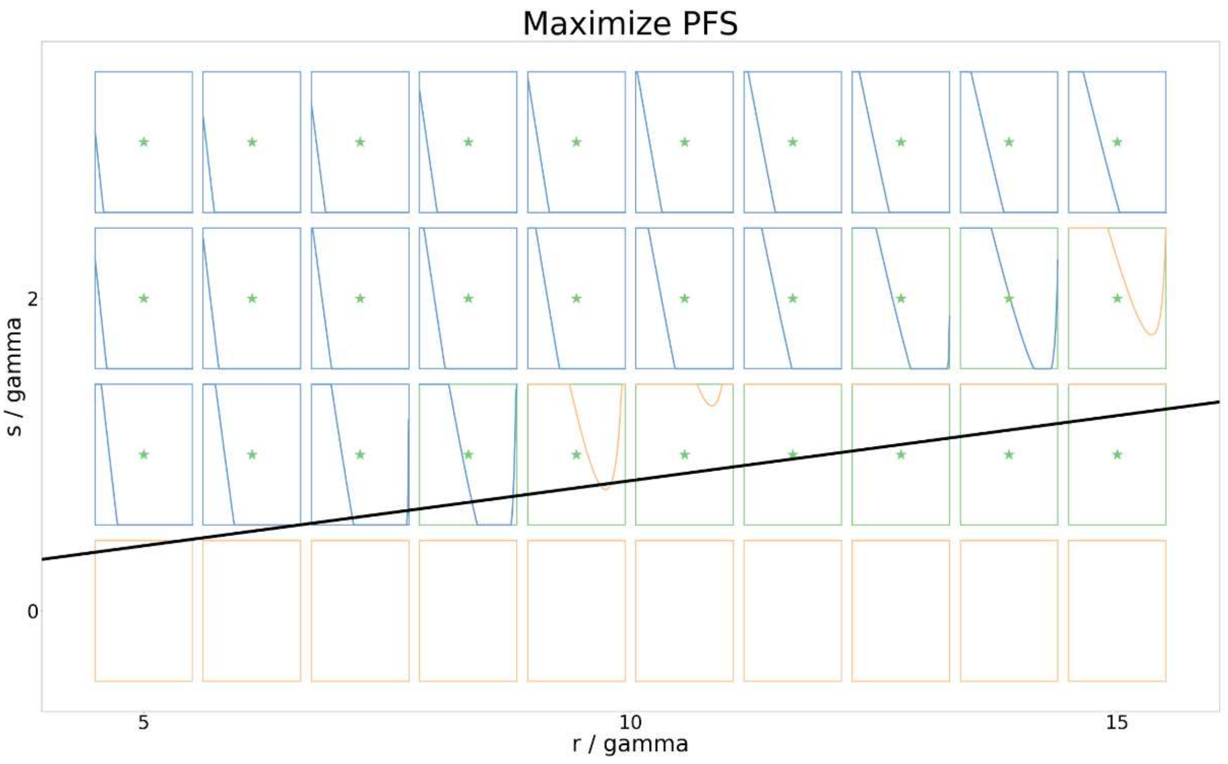

FIG. S7. **Examining the E-type/T-type phase boundary using different curative cycle conditions.** Each box (color coded as in the PFS phase diagram of Fig 3(E)) shows Eq (S13) plotted on the restricted range $[0, 1]$. When it has a root, a curative cycle is possible (blue); otherwise, a curative cycle is not possible (orange). Plots with a green star favor adaptive therapy (delaying a dose gives a PFS benefit). Points above the black line have a curative cycle when infinitesimal switches are allowed, as predicted by Eq (S6); however, some of these schedules become inaccessible due to the discrete dosing cycle time of 7 days.

The change in T over the first leg of the cycle is $dT_1 = (\gamma - \delta E_0)T_0 dt_1$. Over the second leg, it is

$$dT_2 = \gamma T_0 dt_2 = \frac{\gamma T_0 r dt_1}{s(1 - E_0)} \; . \tag{S4}$$

Combining, we find the total change in T is

$$dT = dT_1 + dT_2 = \left[\gamma - E_0\delta + \frac{\gamma r}{s(1 - E_0)}\right] T_0 dt_1 \; . \tag{S5}$$

The sign of dT is determined by the term in brackets in Equation (S5), which is equivalent to a quadratic in E_0. If the quadratic has a root, then the curative cycle is possible. The

corresponding condition is

$$\frac{\delta}{4\gamma}\left(1+\frac{\gamma}{\delta}\right)^2 - 1 > \frac{r}{s}\ , \tag{S6}$$

which gives a linear boundary for the phase diagram, shown as the black line in Fig S7.

However, this curative schedule is not always accessible due to the discretization of time. Infinitesimal switching times are not allowed. The drug must be active for at least 7 days, and then it must be off for at least 7 more days. Without the discretization, every schedule above the black phase boundary of Fig S7 would be exactly E-type and curative, making for a sharp phase boundary (at least on that side; this argument does not comment on the optimal schedule on the other side of the boundary). The chunking of time into weekly cycles blurs the boundary, creating behavior like a second-order phase transition.

Because of this difficulty, we sought another metric to characterize the phase boundary in the case of weekly cycles. This metric is the result of the same calculation as above, but now $dt_i \to \Delta t_i$ becomes a finite time, with $\Delta t_1 = 7$ days. Instead of being able to use Eqs (S1) and (S2), we need the solutions evaluated at Δt_1 and $\Delta t_1 + \Delta t_2$:

$$E(\Delta t_1) = E_0 e^{-r\Delta t_1}\ , \tag{S7}$$

$$T(\Delta t_1) = T_0 e^{\gamma\Delta t_1} \exp\left[\frac{E_0\delta}{r}\left(e^{-r\Delta t_1} - 1\right)\right]\ , \tag{S8}$$

$$E(\Delta t_1 + \Delta t_2) = \frac{E(\Delta t_1)}{E(\Delta t_1) + (1 - E(\Delta t_1))e^{-s\Delta t_2}}\ , \tag{S9}$$

$$T(\Delta t_1 + \Delta t_2) = T(\Delta t_1)e^{\gamma\Delta t_2}\ . \tag{S10}$$

Setting $E(\Delta t_1 + \Delta t_2) = E_0$ allows us to find

$$\Delta t_2 = \frac{1}{s}\ln\left[\frac{1/E(\Delta t_1) - 1}{1/E_0 - 1}\right]\ . \tag{S11}$$

Using this expression for Δt_2 and setting $T(\Delta t_1 + \Delta t_2) < T_0$ gives a condition that must be satisfied for a curative cycle with $\Delta t_1 = 7$ days to exist:

$$T_0 > T_0 \exp\left[\gamma\Delta t_1 + \frac{E_0\delta}{r}\left(e^{-r\Delta t_1} - 1\right) + \frac{\gamma}{s}\ln\left(\frac{e^{r\Delta t_1}/E_0 - 1}{1/E_0 - 1}\right)\right]\ . \tag{S12}$$

Upon dividing by T_0 and taking the natural log, we get

$$0 = \gamma \Delta t_1 + \frac{E_0 \delta}{r} \left(e^{-r\Delta t_1} - 1 \right) + \frac{\gamma}{s} \ln \left[\frac{1 - E_0 e^{-r\Delta t_1}}{(1 - E_0) e^{-r\Delta t_1}} \right] . \quad \text{(S13)}$$

If there exists an E_0 that satisfies this equation, then a curative cycle with $\Delta t_1 = 7$ days is theoretically possible. However, there is no reason Δt_2 should be a multiple of 7 days, and there is no reason why E_0 should be exactly attainable from the simulation's starting value of $E(0) = 1$ (e.g. treating for one week may overshoot E_0).

In Fig S7, we plot the right-hand side of Eq (S13) as E_0 varies from 0 to 1 for each point we tested in phase space. For clarity, we have restricted its range to $[0, 1]$. The color of the curve denotes if a root exists (E-type favored, blue) or not (E-type not favored, orange). The color of the outline box corresponds to the strategy sorting we did in Fig 3(E). Finally, we tested if the PFS time was greater for a schedule that started with two consecutive doses or for a schedule with the second dose delayed until just before progression. Such a set of parameter values would favor an adaptive therapy; these are denoted with green stars. Note that the curative phase boundary line defined by the roots of Eq (S13) is not the same as the one corresponding to Eq (S6) (black line) where infinitesimal cycling is allowed; as expected, the infinitesimal cycle boundary is more permissive, saying that some parameters where we found only T-type adaptive therapy as the optimal schedule actually permit a curative schedule.

In the three anomalous cases (blue curves but green outline boxes), we can see that Eq (S13) does have a root, so that in theory there is a curative E-type schedule. However, this schedule cannot be found because of the discretization of time into 7 day cycles. Accordingly, the best that the algorithm can do in the PFS case is the T-type schedule. In this sense, the phase transition here acts like a second-order phase transition, where properties of both the T-type and E-type schedules can emerge near the boundary, for both optimization objectives.

Our results have largely used fixed values for δ and γ. Eq (S6) tells us directly how the curative cycle boundary (and thus the E-type/T-type phase boundary) shifts as δ and γ change. Unsurprisingly, as shown in Fig S8, decreasing δ or increasing γ makes it more difficult to have a curative cycle, so the region of parameter space where curative cycles are possible gets smaller (the phase boundary shifts upward, toward the s-axis).

Two drugs. In the main text, we analyzed a somewhat special case of the two drug optimization problem where the re-sensitization of the second drug was zero, $s_2 = 0$, and

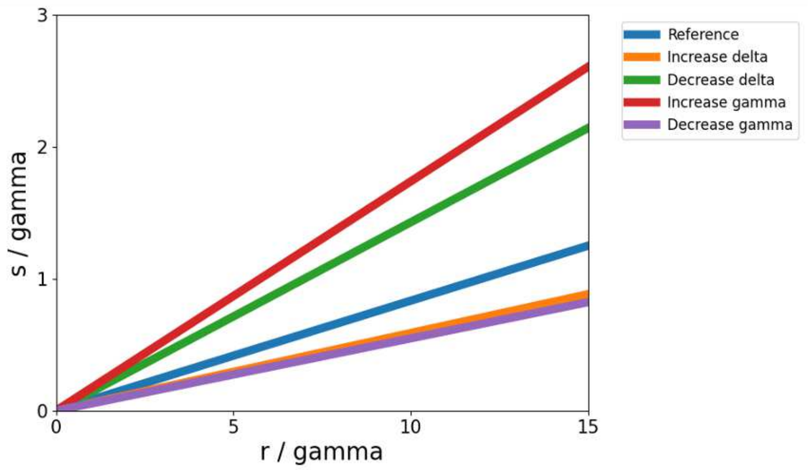

FIG. S8. **Shifting the curative cycle boundary of Eq** (S6) **by varying δ and γ.** Increasing γ or decreasing δ makes it harder to have curative cycles, decreasing the amount of parameter space for which these cycles are possible. Conversely, increasing δ or decreasing γ makes curative cycles more attainable, increasing the size of parameter space over which these cycles are possible.

the efficacy of the second drug is essentially entirely used up in one treatment window. Because of these characteristics, the scheduling optimization problem collapses to the one drug scenario after the use of the second drug (which typically happens very early in the trajectory, during the first few treatment windows). Therefore, it should be possible to use the categories of the one drug solution to categorize the adaptive therapy optimal schedules in the case of maximizing PFS. But now, the analogous schedules would use the second drug as much as possible in the treatment breaks.

The result of this analysis is shown in Fig S9, where the green denotes the T-type analog and blue denotes the rest, including the E-type analog. The phases seem to be quite mixed in this case, with no apparent phase boundary. However, upon inspecting the trajectories (e.g. see the code), this phenomenon can be explained by the second-order-like phase transition we discussed above. The black line marks the (lower) edge of parameter space where curative infinitesimal cycles of drug one are possible, as predicted by Eq (S6). Some of the schedules above have curative cycles in the discrete cycle time case, as shown in Fig 4. The others here either do not, or could not be run long enough in the simulated annealing algorithm to find the curative schedule. In particular, for the green squares that appear above the black line, the T-type adaptive therapies tested were able to achieve much longer times than

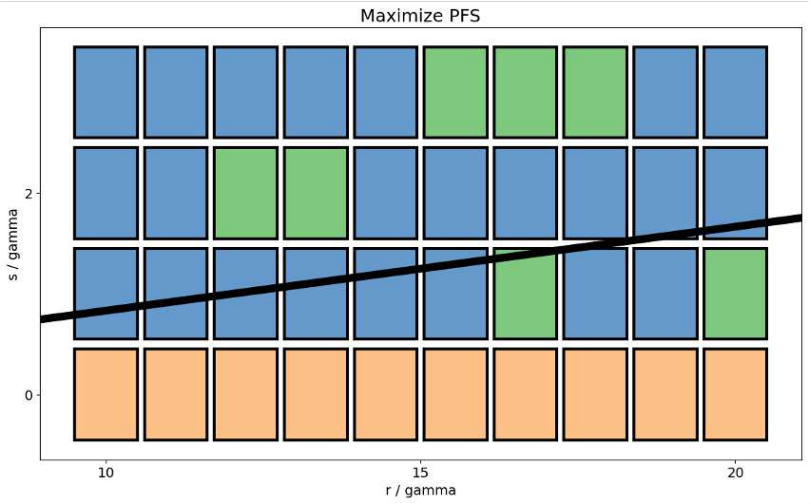

FIG. S9. **Alternate optimal schedule sorting of the two drug phase boundary from Fig 4G.** Because of the special parameter values of the second drug, we were able to map the adaptive therapy schedules onto the one drug case (orange is still the sequential schedule): the T-type analog schedule is shown in green, while all others, including the E-type schedule, are shown as blue. Infinitesimal curative cycles of the first drug exist above the black line, which was calculated using Eq (S6). As before, the second-order nature of the phase boundary causes anomalies and fuzziness, although it is much more pronounced in this case than in Fig S7.

the simulated annealing algorithm could sample. We expect E-type-like schedules to again emerge as the optimum for these cases if the simulated annealing algorithm is permitted to run for much longer.

Below the infinitesimally curative boundary, there are many blue boxes. These points largely correspond to schedules that blend together the E- and T-type strategies, as is the behavior of the second-order phase transition. In the two drug case, the effect is now more pronounced because the use of the second drug creates such a large gap between the tumor population minimum and the progression threshold. So once again, the discretization of time into cycles and the nature of the resulting phase transition explain the anomalous behavior of the phase diagram.

Appendix D: Specific parameters used to create the figures in this paper

We wanted to focus the model on patient-realistic regions of GDRS parameter space. Since the standard of care for Ewing sarcoma is the alternating VDC/IE chemotherapy regimen with two week cycles [30], we used it as the basis for our parameter selection. We set our cycle time smaller, to one week, so we had more flexibility in timing (that is, so doses could be moved around with one week resolution instead of two week resolution). Lung metastases from bone tumors have a doubling time of around 23 days [69], so we generally chose our growth rate $\gamma = \log(2)/20$ to be near this value. We then picked some baseline values for δ, r, and s that created "reasonable" trajectories of the tumor burden, i.e. ones that could create a multitude of responses (non-responders, stable disease, partial response, and complete response, as determined by a tumor detection threshold of $\sim 10^9$ cells). These explorations informed our later choices of parameters that went into creating the figures, as detailed below.

Our simulations were greatly sped up by using the analytical solutions of Eqs (S1) and (S2) in the simulated annealing algorithm instead of using the numerical solution for the computations. We only use the numerical solution for visualization at the end, when we plot both it and the analytical solution; e.g. see Fig S5, where the analytical solution is denoted by the black stars. We will now explain how this solution was utilized in the algorithm.

The analytical solutions to Eqs (S1) and (S2) are

$$T(t) = \begin{cases} T_0 e^{\gamma t} \ , & \text{drug off} \\ T_0 e^{\gamma t} \exp\left[\frac{E_0 \delta}{r}\left(e^{-rt} - 1\right)\right] \ , & \text{drug on} \end{cases} \ , \tag{S1}$$

$$E(t) = \begin{cases} \frac{E_0}{E_0 + (1 - E_0) e^{-st}} \ , & \text{drug off} \\ E_0 e^{-rt} \ , & \text{drug on} \end{cases} \ , \tag{S2}$$

where, for the purposes of the simulation, drug is on or off for the entire cycle period and T_0 and E_0 are known from the previous cycle (that is, $t = 0$ is the current cycle start time). So as not to clutter the equations with indices, these equations assume only one drug. For more drugs, each $E_i(t)$ would need to be tracked for each cycle, using its own E_{i0}, δ_i, r_i, and s_i, as well as the drug on or off solution corresponding to whether that drug was on or off during that cycle. The full solution for the entire duration of the treatment is a piecewise

combination of these individual cycle solutions arranged and solved according to the drug schedule.

These analytical functional forms allow for easy evaluation of the values of T and E at the end of the cycle. However, we also need to know the minimum and maximum values that T attains during the period to check for progression or cure. The maximum value of T will always occur at the cycle start or end, so no additional calculations were needed to find the maximum. The minimum, however, can occur at an intermediate point when drug is on (due to resistance). There is an intermediate minimum if the sign of $\delta - \gamma E(t)$ changes from negative at the start of the cycle to positive at the end of the cycle. If this condition is satisfied, then the time at which the minimum occurred was

$$t_{\text{minimum}} = \frac{1}{r} \log\left(\frac{\delta E_0}{\gamma}\right) , \tag{S3}$$

where again $t = 0$ is set to be the cycle start time. Then, the intermediate values of T and E could be solved for using Eqs (S1) and (S2).

In addition to checking for and returning the values of the intermediate minima, we also stopped the dynamics (just returned the previous value every subsequent cycle) if the patient progressed or was cured. The patient was checked for progression or cure that occurred at intermediate times as well to achieve this goal. This set of rules explains the dynamics of the analytical solution black stars for every plot in which they appear. For full details of the implementation, see the function "GDRSanalytical" in the "schedule_utils.py" file of the code provided on GitHub (see code availability statement in the main text).

Finally, before we give a detailed account of each figure, we revisit the cooling schedule used throughout. The cooling schedule of Eq (4) is compared to the energy of the simulation in Fig S10. This cooling schedule allowed for a period of exploration (temperature higher than energy) and a period of exploitation (temperature lower than energy), giving generally good convergence near the global optimum for every parameter set tested.

Figure 1. For these figures, we used $\gamma = \log(2)/20$, $\delta_1 = \delta_2 = \delta_3 = 30\gamma$, $r_1 = r_2 = r_3 = 4\gamma$, and $s_1 = s_2 = s_3 = 0$. We then numerically simulated the GDRS model for the shown schedules of drug on/off.

Figure 3. (A) We found this continuous schedule to be optimal for $\gamma = \log(2)/20$, $\delta = 30\gamma$, $r = 2\gamma$, and $s = 0$ when trying to minimize the tumor burden. (B) We found this E-type adaptive therapy schedule to be optimal for $\gamma = \log(2)/20$, $\delta = 25\gamma$, $r = 6\gamma$,

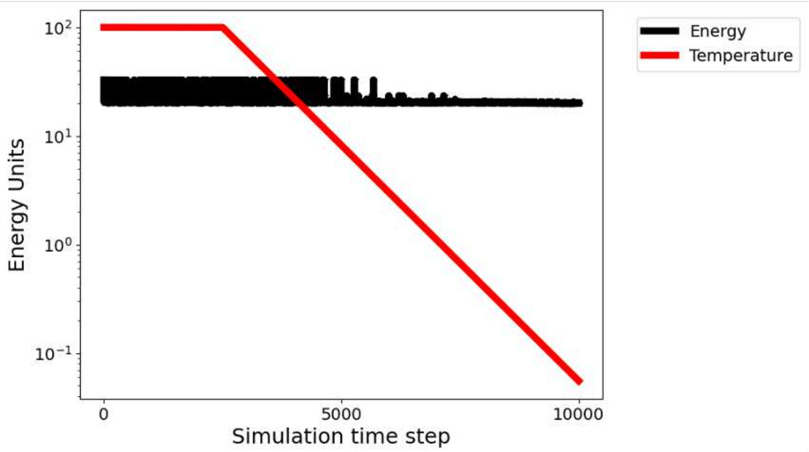

FIG. S10. **The cooling schedule used for the simulated annealing algorithm, Eq** (4)**,** demonstrated here using the simulation results from Fig S6.

and $s = 7\gamma$ when trying to minimize the tumor burden. (C) We found this T-type adaptive therapy to be optimal for $\gamma = \log(2)/20$, $\delta = 25\gamma$, $r = 6\gamma$, and $s = \gamma$ when trying to maximize PFS. (D and E) With $\gamma = \log(2)/20$ and $\delta = 50\gamma$, we sampled 40 points in $r - s$ space, evenly spaced between $r = 5\gamma$ to 15γ and $s = 0$ to 3γ for each objective.

Figure 4. (A) We found this sequential MTD schedule to be optimal for $\gamma = \log(2)/20$, $\delta_1 = \delta_2 = 40\gamma$, $r_1 = 6\gamma$, $r_2 = 10\gamma$, and $s_1 = s_2 = 0$ when trying to maximize PFS. (B) We found this alternating schedule to be optimal for $\gamma = \log(2)/20$, $\delta_1 = 50\gamma$, $\delta_2 = 30\gamma$, $r_1 = 3\gamma$, $r_2 = 2\gamma$, and $s_1 = s_2 = 0$ when trying to maximize the PFS. (C) We found this second-strike therapy to be optimal for $\gamma = \log(2)/20$, $\delta_1 = 50\gamma$, $\delta_2 = 40\gamma$, $r_1 = 18\gamma$, $r_2 = 4\gamma$, $s_1 = \gamma$, and $s_2 = 0$ when trying to minimize the tumor burden. (D) We found this curative adaptive therapy to be optimal for $\gamma = \log(2)/20$, $\delta_1 = 50\gamma$, $\delta_2 = 40\gamma$, $r_1 = 10\gamma$, $r_2 = 4\gamma$, $s_1 = 2\gamma$, and $s_2 = 0$ when trying to minimize the tumor burden. (E) We found this non-curative adaptive therapy to be optimal for $\gamma = \log(2)/20$, $\delta_1 = 50\gamma$, $\delta_2 = 40\gamma$, $r_1 = 18\gamma$, $r_2 = 4\gamma$, $s_1 = \gamma$, and $s_2 = 0$ when trying to maximize the PFS. (F and G) With $\gamma = \log(2)/20$, $\delta_1 = 50\gamma$, $\delta_2 = 40\gamma$, $r_2 = 7\gamma$, and $s_2 = 0$, we sampled 40 points in $r_1 - s_2$ phase space, evenly spaced between $r_1 = 10\gamma$ to 20γ and $s_1 = 0$ to 3γ for each objective.

Figure S4. For the example schedule maximizing the PFS, we used $\gamma = \log(2)/20$, $\delta = 25\gamma$, $r = 15\gamma$, and $s = \gamma/2$. For the example schedule minimizing the tumor burden, we used $\gamma = \log(2)/20$, $\delta = 50\gamma$, $r = 6\gamma$, and $s = 45\gamma/4$.

Figure S5. Here we minimized the tumor burden using $\gamma = \log(2)/20$, $\delta = 10\gamma$, $r = 2\gamma$,

and $s = 10\gamma$.

Figure S6. Here we maximized the PFS using $\gamma = \log(2)/20$, $\delta = 10\gamma$, $r = 3\gamma$, and $s = \gamma/2$.

Figure S7. We used the same values as for Figure 3(E), plotting the additional equations as described above.

Figure S8. Here we plotted $r(s)$ for $s = 0$ to $3\log(2)/20$ using Eq (S6) for the following various parameter combinations. Reference: $\gamma = \log(2)/20$, $\delta = 5\log(2)/2$. Increase delta: $\gamma = \log(2)/20$, $\delta = 7\log(2)/2$. Decrease delta: $\gamma = \log(2)/20$, $\delta = 3\log(2)/2$. Increase gamma: $\gamma = \log(2)/10$, $\delta = 5\log(2)/2$. Decrease gamma: $\gamma = \log(2)/30$, $\delta = 5\log(2)/2$.

Figure S9. We used the same parameters as Fig 4(G).
